# Substrate Specificity of the *Bacillus subtilis* BY-Kinase PtkA Is Controlled by Alternative Activators: TkmA and SalA

**DOI:** 10.3389/fmicb.2016.01525

**Published:** 2016-09-26

**Authors:** Abderahmane Derouiche, Lei Shi, Aida Kalantari, Ivan Mijakovic

**Affiliations:** ^1^Division of Systems and Synthetic Biology, Department of Biology and Biological Engineering, Chalmers University of TechnologyGothenburg, Sweden; ^2^Novo Nordisk Foundation Center for Biosustainability, Technical University of DenmarkLyngby, Denmark

**Keywords:** bacterial protein-tyrosine kinase, protein phosphorylation, kinase specificity, kinase activator, transcription factor

## Abstract

Bacterial protein-tyrosine kinases (BY-kinases) are known to regulate different aspects of bacterial physiology, by phosphorylating cellular protein substrates. Physiological cues that trigger BY-kinases activity are largely unexplored. In *Proteobacteria*, BY-kinases contain a cytosol-exposed catalytic domain and a transmembrane activator domain in a single polypeptide chain. In *Firmicutes*, the BY-kinase catalytic domain and the transmembrane activator domain exist as separate polypeptides. We have previously speculated that this architecture might enable the *Firmicutes* BY-kinases to interact with alternative activators, and thus account for the observed ability of these kinases to phosphorylate several distinct classes of protein substrates. Here, we present experimental evidence that supports this hypothesis. We focus on the model *Firmicute-*type BY-kinase PtkA from *Bacillus subtilis*, known to phosphorylate several different protein substrates. We demonstrate that the transcriptional regulator SalA, hitherto known as a substrate of PtkA, can also act as a PtkA activator. In doing so, SalA competes with the canonical PtkA activator, TkmA. Our results suggest that the respective interactions of SalA and TkmA with PtkA favor phosphorylation of different protein substrates *in vivo* and *in vitro*. This observation may contribute to explaining how specificity is established in the seemingly promiscuous interactions of BY-kinases with their cellular substrates.

## Introduction

BY-kinases are bacterial enzymes found in roughly one half of sequenced bacterial species, but with no direct counterparts in *Eukarya* (Jadeau et al., 2008; Shi et al., 2014a). Their function is to regulate different cellular processes via phosphorylation of protein substrates. It is important to note that a single BY-kinase can phosphorylate several distinct proteins, and affect their respective roles. For example, the model BY-kinase PtkA from *Bacillus subtilis* regulates the activity of several enzymes (Mijakovic et al., 2003; Petranovic et al., 2009; Jers et al., 2010) and protein chaperones (Shi et al., 2016). In addition, PtkA has a role in regulating biofilm development (Kiley and Stanley-Wall, 2010; Gerwig et al., 2014), DNA metabolism (Petranovic et al., 2007), and sub-cellular localization of proteins (Jers et al., 2010). Finally, PtkA also phosphorylates transcriptional regulators and controls their binding to DNA target sequences (Derouiche et al., 2013, 2015). For a global overview of various regulatory roles of BY-kinases, several reviews are available (Grangeasse et al., 2007, 2012; Chao et al., 2014; Mijakovic and Deutscher, 2015; Mijakovic et al., 2016). In *Proteobacteria*, such as *Escherichia coli*, BY-kinases are large transmembrane proteins. They contain a catalytic domain exposed to the cytosol, and an activator domain, typically with a flexible extracellular loop flanked by two transmembrane helices (Doublet et al., 2002). In *Firmicutes*, such as *B. subtilis*, the BY-kinase domain is in most cases a self-standing protein that engages in protein–protein interaction with a separate transmembrane activator (Mijakovic et al., 2003). The *Firmicute*-type BY-kinase and activator pairs are typically encoded by neighboring genes, and this has sparked a long-standing debate on whether the prototype BY-kinase was of the *Firmicute* (“split”) or *Proteobacteria* (“joint”) type (Grangeasse et al., 2007; Shi et al., 2010). We have previously speculated that the rationale for the “split” type architecture could be to enable the BY-kinase domain to interact with more than one activator (Shi et al., 2010). First evidence in this direction came from the study by Shi et al. (2014c), demonstrating that autophosphorylation of PtkA can be stimulated in the presence of the cell division protein MinD *in vitro*. That study had, however, not established whether MinD-dependent activation of PtkA can lead to specific phosphorylation of any of the PtkA substrates. An interesting point is that PtkA and MinD share a large extent of structural homology (Derouiche et al., 2016). Their ATP-binding motifs belong to the family of domains distinguished by the conserved motifs known as Walker A, A’ and B (Walker et al., 1982). Curiously, another *B. subtilis* protein bearing Walker motifs is functionally related to PtkA. The PtkA/MinD homolog SalA, belonging to the Mrp ATPase family, had been originally described as an indirect positive regulator of the expression of the exoprotease AprE (Ogura et al., 2004). We have recently established that SalA is a transcriptional regulator, which binds upstream of the *scoC*, thus repressing this repressor of *aprE* (Derouiche et al., 2015). We have also shown that SalA directly interacts with the BY-kinase PtkA, which results of phosphorylation of SalA Y327, situated in the C-terminal domain. This phosphorylation activates the inter-related ATP- and DNA-binding functions of SalA, and it ultimately leads to repression of *scoC* and overexpression of *aprE in vivo* (Derouiche et al., 2015).

In this study, we present evidence that SalA is not only a substrate of PtkA, but also its activator. The interaction between SalA and PtkA leads to mutual regulation: phosphorylation of SalA as previously documented and activation of the kinase function of PtkA, as demonstrated in this report. SalA acts as an alternative to the canonical PtkA activator, TkmA. The genes encoding the kinase PtkA and the activator TkmA are adjacent, in the same operon (Mijakovic et al., 2003). TkmA and SalA both lead to stronger kinase autophosphorylation, but they seem to have different effects when it comes to PtkA substrate phosphorylation. Our results point to the existence of different classes of PtkA substrates: those phosphorylated preferentially in the presence of TkmA, those phosphorylated preferentially in the presence of SalA, and those phosphorylated equally in the presence of either activator. This finding could be a useful basis for deciphering the specificity in complex interaction networks of BY-kinases and their substrates.

## Materials and Methods

### Bacterial Strains and Growth Conditions

For gene cloning, *E. coli* NM522 was used. The strain *E. coli* M15, carrying pREP4-GroESL (Amrein et al., 1995), was used for biosynthesis of tagged proteins. For *in vivo* mutant construction, *B. subtilis BS514* (*B. subtilis* 168 *trp^+^ Pr:: neoR)* was used. All mutants used in this work are listed in the Supplementary Table S1. *B. subtilis* and *E. coli* strains were grown in Luria-Bertani (LB) medium with shaking, at 37°C. When relevant, ampicillin (100 μg/ml) and kanamycin (25 μg/ml) for *E. coli* and erythromycin (1 μg/ml), neomycin (5 μg/ml), and phleomycin (2 μg/ml) for *B. subtilis* were added to the medium.

### DNA Manipulation and Strain Construction

All PCR primers with restriction enzymes are listed in Supplementary Table S2. The deletion of *tkmA* gene was performed using the modified mutation delivery method of Fabret et al. (2002). The deletion of *tkmA* gene was made using the pairs of primers ΔtkmA-ext fwd/ΔtkmA-ext rev were used. To introduce an in-frame deletion between the codons 10 and 240 of the gene *tkmA*, partially complementary primers Δ*tkmA fwd*/Δ*tkmA* rev were used. Gene *fatR* was amplified by PCR from *B. subtilis* 168 genomic DNA and inserted into the vector pSG1729 (Lewis and Marston, 1999) between the restriction enzymes site *KpnI* and *XhoI*, which results in the replacement of the *gfp* gene by *Strep-fatR. B. subtilis* wild type (WT). Δ*salA* (Derouiche et al., 2015), Δ*ptkA* (Jers et al., 2010), and Δ*tkmA* strains were transformed with the *pSG1729-fatR* construct and selected for erythromycin resistance. All constructs were sequenced to check for absence of unsolicited mutations.

### Synthesis and Purification of Tagged Proteins

SalA, Ugd, Asd, and FatR proteins were produced in *E. coli* M15 as 6x His N-terminal fusions. The cultures were grown with shaking (200 rpm) at 37°C until OD_600_ of 0.5. To induce the expression, 1 mM of isopropyl β-D-1 thiogalactopyranoside (IPTG) was added to the medium, and the cultures were kept in the same growth conditions for three more hours. To purify the 6x His-tagged proteins, Ni-NTA columns were used (Qiagen) as described previously (Mijakovic et al., 2003). The Step- tagged FatR protein was purified from *B. subtilis BS514* using the Strep Tactin affinity chromatography (Novagen) as described in our previous study (Shi et al., 2014b). The purity of proteins was checked by SDS-PAGE and aliquots of all purified proteins were kept at -80°C in buffer composed of 50 mM Tris-Cl pH 7.5, 100 mM NaCl and 10% glycerol.

### *In vitro* Protein Phosphorylation Assays

All *in vitro* phosphorylation assays were performed in the presence of 50 μM ATP [γ-^32^P], which corresponds to 20 μCi/mmol of labeled ATP. A standard 40 μl reaction mix contained 1 μM BY-kinase PtkA and 1 μM activator proteins (TkmA-NCter or SalA). Concentration of protein substrates was 5 μM, and the reaction medium contained 1 mM MgCl_2_ and 100 mM Tris-HCl (pH 7.5). After 1 h of incubation at 37°C, the reactions were stopped by adding SDS-PAGE loading dye and heating for 5 min at 100°C. The proteins were separated by electrophoresis using SDS-PAGE. The gels were washed and then boiled in 0.5 M HCl for 10 min, and transient staining with Coomassie Blue was used to identify protein bands. The gels were dried overnight and the autoradiography signals of phosphorylated proteins were revealed by a PhosphoImager (FUJI). The experiments were performed in triplicates, each time with protein aliquots purified independently. One representative assay is shown for each reaction.

### Asd and Ugd Assay in Crude Extracts

For enzymatic assays, *B. subtilis* strains were grown in LB with vigorous shaking in flasks at 37°C. Samples were harvested at the mid-exponential phase (OD_600_ = 0.4). Cells were lysed by sonication. The lysis buffers (composition adapted for each assay) contained 1% tyrosine phosphatase inhibitor cocktail (Sigma) and 1 mg/ml lysozyme (Sigma). For the Ugd assay, the buffer contained 50 mm Tris-HCl pH 7.5, 50 mm NaCl and 10% glycerol. For the Asd assay, the buffer contained 50 mM 3-(N-morpholino) propanesulfonic acid (pH 7.0), 200 mM KCl, 0.1 mM EDTA, and 10 mM 2-mercaptoethanol. After centrifugation, the supernatant was desalted on PD-10 columns (GE healthcare), to remove all metabolites and cofactors. Total protein concentration was standardized using the Bradford assay. The Asd assay was performed with 100 μg of total protein, as described by Jers et al. (2010). The only difference was that instead of adding commercially available aspartate kinase, the aspartate kinases present in the crude extract of *B. subtilis* (Graves and Switzer, 1990; Roten et al., 1991; Kobashi et al., 2001) were used to convert ATP and aspartate to aspartyl phosphate. The Ugd assay was performed with 100 μg of total protein, as described by Pagni et al. (1999).

### Far-Western Blot

To examine competition between SalA and TkmA for PtkA binding, purified TkmA was separated on a 12 % SDS-PAGE gel. Protein content of the gel was transferred to a PVDF membrane using a *Trans*-blot cell from Bio-Rad. The transfer buffer (pH 8.3) was composed of 25 mM Tris, 125 mM Glycine, and 10% ethanol. The membrane was washed three times with 50 ml MQ water and then blocked overnight (at 4°C) with 5% BSA in a buffer containing 25 mM Tris pH 8, 125 mM NaCl, and 1% Tween 20. The assay was carried out with 20 nM Strep-tagged PtkA alone, or mixed with SalA in different ratios (indicated in the figure legend). PtkA (and SalA where indicated) were added to the membrane, and the mix was incubated for 1 h at room temperature. To detect the interaction between PtkA and TkmA, the membrane was incubated with 1:1000 of conjugate Srep-tactin HRP (IBA Biotechnology) in 1% BSA for 1 h in TBS-T buffer as described previously (Derouiche et al., 2015). The chemiluminescence signals were visualized using an AEC chromagen kit (SIGMA). Three replicates were performed with independently purified proteins, and one representative experiment is shown.

### Electrophoretic Mobility Shift Assays

Strep-tagged FatR was purified from *B. subtilis* in different backgrounds: WT, Δ*salA*, and Δ*tkmA*. The DNA binding assay was performed with a 24 bp double-stranded DNA containing the *fatR* operator sequence, as described by Derouiche et al. (2013). The molar ratio of the DNA probe to strep-tagged FatR proteins is indicated in the figure legend. The reaction mixtures were incubated for 1 h at the room temperature. The migration was performed for 2 h at 2 V/cm in 0.5 Tris-acetate-EDTA using non-denaturing gels (12% polyacrylamide). The experiment was repeated three times with independently purified proteins. One representative experiment is shown.

## Results and Discussion

### SalA Is an Activator of PtkA

**FIGURE 1:**
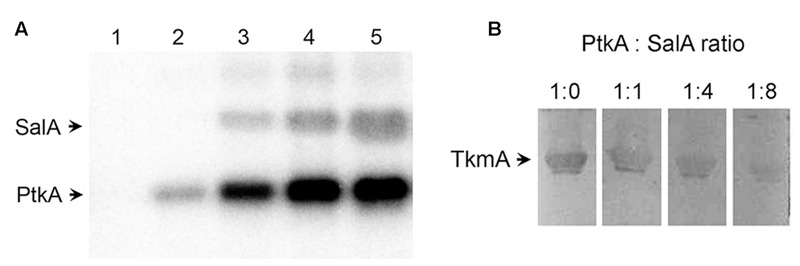
**SalA activates PtkA and competes with TkmA for interaction with PtkA. (A)** *In vitro* phosphorylation assay with purified SalA and PtkA. All lanes except number 1 contained 1 μM PtkA. SalA concentration was 1 μM in lane 1, it was absent in lane 2, and its concentration was 0.25, 0.5, and 1 μM in lanes 3–5, respectively. Reactions were incubated for 60 min with ^32^P-γ-ATP, and run on SDS-PAGE, and the signals were visualized by autoradiography. **(B)** Far-Western analysis of TkmA/PtkA interaction in the presence of different concentrations of SalA. Four identical samples, each containing 20 μg of TkmA, were separated on SDS-PAGE and blotted onto a PVDF membrane. Lanes were cut in individual strips and each was incubated for 2 h with 12 μM Strep-tagged PtkA, mixed with different molar ratios of SalA (as indicated above each lane). The TkmA-PtkA interaction was detected using the Strep-tactin HRP conjugate.

Recently, we have established that the C-terminus of SalA can interact directly with the BY-kinase PtkA, which results in phosphorylation of the SalA residue tyrosine 327. This phosphorylation enhances its function as a repressor of *scoC* (Derouiche et al., 2015). Interestingly, the *in vitro* phosphorylation of SalA took place in absence of the canonical activator TkmA (Derouiche et al., 2015). This finding is difficult to reconcile with the available structural data. The BY-kinases from *Firmicutes* require the interaction with TkmA-type activators to stabilize the ATP binding pocket in their active site in order to autophosphorylate or phosphorylate substrates (Olivares-Illana et al., 2008). The presence of TkmA is a strict requirement for phosphorylation of all previously characterized PtkA substrates (Mijakovic et al., 2003, 2006; Jers et al., 2010; Derouiche et al., 2013, 2015; Shi et al., 2016). The first clue in explaining this observation comes from the structural homology of SalA with BY-kinases and MinD proteins (Derouiche et al., 2016). A part of the C-terminal region of SalA which interacts with PtkA shows sequence homology (by circular permutation) with the activating fragment of the *Staphylococcus aureus* BY-kinase activator CapA and the N-terminus of its cognate BY-kinase CapB (Olivares-Illana et al., 2008). This suggested that the C-terminus of SalA could be expected to activate PtkA. To investigate this, we performed a series of PtkA autophosphorylation reactions, keeping the kinase concentration constant and varying the concentration of SalA (**Figure 1A**, lanes 2–5). The *in vitro* phosphorylation assay demonstrated that the presence of SalA strongly stimulates autophosphorylation of PtkA in the absence of its canonical activator TkmA.

We recently proposed a model for PtkA-SalA kinase-substrate interaction that may take the form of a hetero-octamer in which some PtkA subunits have been replaced with SalA (Derouiche et al., 2015). In this PtkA-SalA interaction model, both the SalA phosphorylated residue Y327, and the SalA sequence homologous to the CapA activator domain, face the PtkA active site. Therefore, the interaction model is consistent with the notion that SalA may activate PtkA. Examination of all available structures and structural models indicates that the interactions of TkmA-type activators and SalA with the active site of the BY-kinase are similar and thus structurally overlapping. Thus, TkmA and SalA should not be able to interact with PtkA simultaneously. To verify this, we performed a Far Western experiment, in which we detected the interaction of TkmA and PtkA. The concentrations of TkmA and PtkA in the assay were fixed, and we varied the concentration of SalA. A clear inhibition of the TkmA-PtkA interaction was observed with higher concentrations of SalA, indicating that SalA competes with TkmA for PtkA binding (**Figure 1B**).

### SalA and TkmA act as Alternative Activators of PtkA and Can Direct the Activity of the Kinase Toward Different Substrates

**FIGURE 2:**
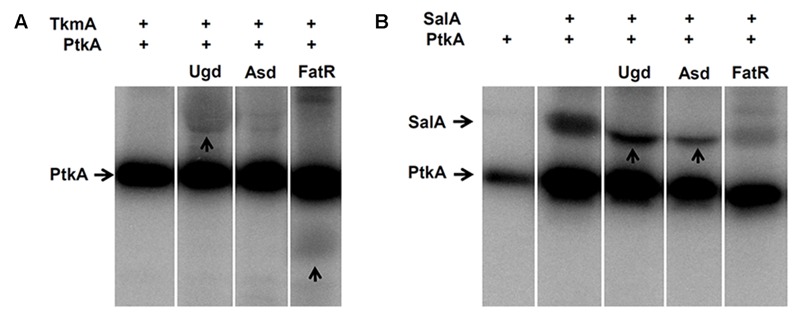
**SalA and PtkA confer different substrate specificities to PtkA. (A)** *In vitro* phosphorylation assay with purified TkmA, PtkA and the PtkA substrates; Ugd, Asd, and FatR. Presence of 1 μM PtkA and 1 μM TkmA is indicated (+) above each lane. Substrate concentration was 5 μM, and the identity of substrate is indicated above each lane. Reactions were incubated for 60 min in the presence of ^32^P-γ-ATP, separated on SDS-PAGE, and the signals were revealed by autoradiography. **(B)** The same type of *in vitro* phosphorylation assay performed with purified SalA, instead of TkmA. Presence of 1 μM PtkA and 1 μM is indicted by (+) above each line, the substrate (indicated above each lane) concentration was 5 μM. For each figure **(A,B)**, all the cut lanes come from the identical gel, and they were not differentially treated by any kind of image processing.

The results presented in the previous section establish SalA as a *bona fide* candidate for an alternative activator of PtkA. If PtkA can interact with either of the two activators *in vivo*, and the activation effect is similar, what is the reason for the existence of two activators? To address this question, an *in vitro* phosphorylation assay with purified proteins was performed, where we compared the ability of PtkA to phosphorylate several characterized protein substrates in the respective presence of either TkmA or SalA (**Figure 2**). The first characterized PtkA substrate, Ugd (Mijakovic et al., 2003; Petranovic et al., 2009), was phosphorylated in the presence of either of the activators. By contrast, SalA promotes the phosphorylation of the PtkA substrate Asd more efficiently (Jers et al., 2010), while TkmA promoted the phosphorylation of the PtkA substrate FatR more efficiently (Derouiche et al., 2013). When SalA served as an activator in the *in vitro* reaction, for example in the reaction with Asd and Ugd, phosphorylation of SalA was abolished (**Figure 2B**, lanes “Asd” and “Ugd”). This could mean that the PtkA-SalA interaction exists in two different and mutually exclusive modes: kinase-substrate and kinase-activator. It would be very interesting from the structural and functional perspective to explore this possibility in the future.

The hypothesis that SalA and TkmA may determine the choice of substrate preferentially phosphorylated by PtkA was next assessed *in vivo*. We used the same three PtkA substrates: Asd (preferentially phosphorylated in the presence of SalA), FatR (preferentially phosphorylated in the presence of TkmA), and Ugd (equally phosphorylated in the presence of either TkmA or SalA). PtkA-dependent phosphorylation is known to lead to a measurable modification of activity of all these substrates: increase of the Asd enzymatic activity (Jers et al., 2010), decrease in the DNA binding affinity of FatR (Derouiche et al., 2013) and increase of the Ugd enzymatic activity (Mijakovic et al., 2003; Petranovic et al., 2009). To test the impact of SalA and TkmA on the phosphorylation of PtkA substrates *in vivo*, we constructed strains with seamless deletions of *salA* and *tkmA*.

**FIGURE 3:**
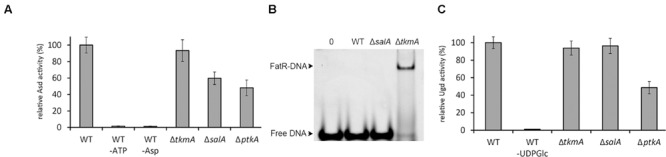
**SalA and TkmA as alternative activators of PtkA. (A)** Aspartate semialdehyde dehydrogenase assay with *B. subtilis* desalted crude extracts from different strains (indicated below each bar). Relative extract activity is expressed as % of activity with respect to the WT strain extract, normalized for total protein concentration. Negative controls include reactions without either ATP or aspartate. The results represent the mean value from three biological replicates. **(B)** Gel-shift experiment with Strep-tagged FatR purified from different *B. subtilis* strains (indicated above each lane), using the FatR operator sequence. In all reactions 20 pmol of DNA target were used in the samples. Lane 1 is a negative control with no protein added, and lanes 2–4 contained 3 nmol of FatR purified from the strains as indicated. The samples were separated in native electrophoresis conditions and DNA was visualized after ethidium bromide staining. The arrows indicate the signal of the free DNA and of the FatR-DNA complex. A representative result from three biological replicates is shown. **(C)** UDP-glucose dehydrogenase assay with *B. subtilis* desalted crude extracts from different strains (indicated below each bar). Relative extract activity is expressed as % of activity with respect to the WT strain extract, normalized for total protein concentration. The negative control is a reaction without UDP-glucose. The results represent the mean value from three biological replicates.

The Asd activity was measured directly in desalted crude extracts with phosphatase inhibitors (to preserve the Asd phosphorylation state). Compared to the WT, the activity was significantly reduced in the Δ*ptkA* and Δ*salA* strains, but not in Δ*tkmA* (**Figure 3A**). This indicated that Asd *in vivo* activation via phosphorylation depends on PtkA and SalA, but not on TkmA. For assessing FatR activity, we prepared *B. subtilis* strains expressing Strep-tagged FatR in the same relevant genetic backgrounds: WT, Δ*salA*, and Δ*tkmA*. FatR was purified in the presence of phosphatase inhibitors and assayed for its ability to bind its target DNA (**Figure 3B**). The binding was not detectable with FatR purified from the WT and Δ*salA* strains, but was detectable with the equal amount of FatR purified from the Δ*tkmA* strain, indicating that the absence of TkmA coincided with less efficient phosphorylation *in vivo*. Finally, for Ugd we also used the enzyme activity essay with desalted crude extracts (with phosphatase inhibitors) (**Figure 3C**). In this case the activity was reduced in Δ*ptkA* compared to the WT, but was not significantly affected in either Δ*salA* or Δ*tkmA*, suggesting that in this case the two activators can complement each other’s absence. Thus all the *in vivo* and *in vitro* findings were in agreement, suggesting that SalA and TkmA can indeed play the role of alternative PtkA activators, with a potential to preferentially direct the kinase activity to certain substrates. Interestingly, our two-hybrid interaction assay had previously revealed that TkmA interacts with PtkB, a second BY-kinase from *B. subtilis* (Shi et al., 2014c). It is therefore tempting to speculate that PtkB might also have two alternative activators: TkmA and TkmB.

### A Mutual Regulatory Loop between PtkA and SalA

**FIGURE 4:**
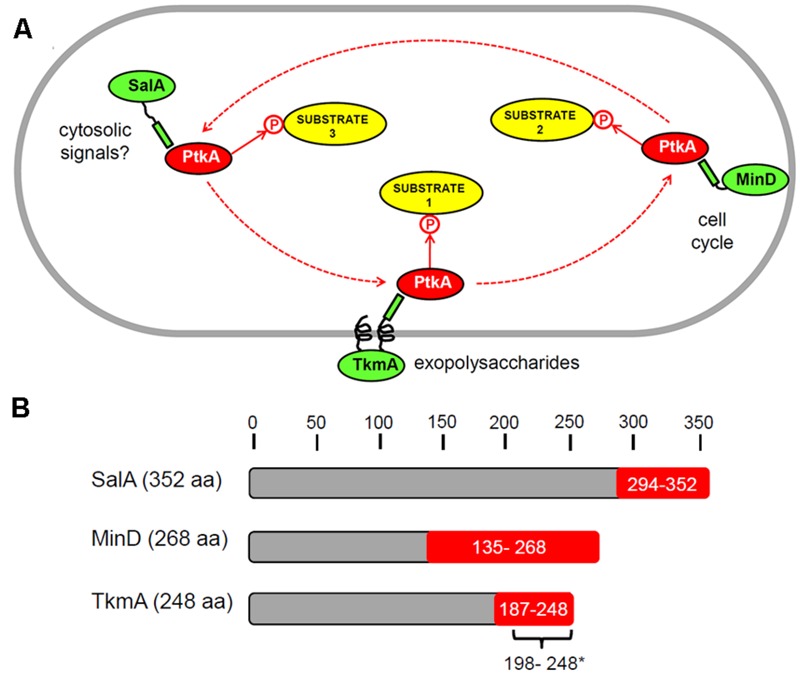
**Schematic illustration of our working hypothesis regarding multiple activators of PtkA. (A)** Different activators: TkmA, SalA, and MinD (in green) compete in the cell for the interaction with the kinase PtkA (red). A certain type of activator, when in complex with the kinase, directs its activity toward a different type of substrate or a sub-set of substrates. The known PtkA activators have different localizations: TkmA – membrane, MinD – cell poles (distribution changes during the cell cycle), SalA – cytosolic. This representation should by no means be taken as a mechanistic model; it is merely a visualization of the concept that PtkA may cycle among different activators in the cell. **(B)** Schematic representation of the domains of the *B. subtilis* proteins TkmA, SalA, and MinD known to interact with PtkA. The minimal interaction domains, shown in red with, were identified in our previous study using yeast two hybrid (Shi et al., 2014c). In addition, the minimal domain of TkmA required for activation of PtkA (^∗^) was determined to comprise the residues 198 to 248 in a biochemical study by Mijakovic et al. (2003).

Our findings indicate that there exists a mutual regulation between *B. subtilis* PtkA and SalA, which critically depends on the physical interaction between the C-terminal region of SalA and the catalytic domain of PtkA (**Figure 4**). This interaction has regulatory consequences for both proteins. In contact with PtkA substrates, SalA activates the kinase but does not get phosphorylated itself. In the absence of other substrates, as described previously (Derouiche et al., 2015), SalA gets phosphorylated by PtkA, this promotes its ATP binding, and in turn stimulates binding to its target DNA sequence, upstream of *scoC*. Accounts of such mutual regulatory loops are not very common in the literature. One example of negative feedback involves the human ubiquitin E3 ligase SIAH2 which is phosphorylated and activated by the serine/threonine kinase DYRK2. In turn, SIAH2 destabilizes and promotes degradation of the kinase (Pérez et al., 2012). Forward-feeding activation loops are exemplified by the murine Src family kinases, which activate the neurotrophin receptor tyrosine kinase TrkB, which in turn activates the kinases (Huang and McNamara, 2010). To the best of our knowledge, this is the first report of a mutual regulation loop based on a direct protein–protein interaction in bacteria. The case is particularly interesting since the interactants share a common evolutionary origin (Derouiche et al., 2016), suggesting that the interaction of SalA and PtkA with mutual regulatory consequences was maintained in the process of divergent evolution. One may expect that such cases of mutual regulation will turn out not to be uncommon in bacteria, since the reports on “moonlighting” proteins which have more than one known function are steadily accumulating (Jeffery, 1999; Wang et al., 2013; Wang and Jeffery, 2016).

## Conclusion

The findings presented here suggest that the access of PtkA to alternative activators: TkmA, SalA or MinD, could determine when and where PtkA acts, and which substrates it phosphorylates. We have previously shown that overexpression of TkmA confines PtkA to the membrane (Jers et al., 2010) and that MinD is capable of recruiting PtkA to the cell pole (Shi et al., 2014c). Therefore, the sub-cellular localization of PtkA may indeed be dynamic, and respond to expression levels or the availability of alternative activators (**Figure 4**). So far no specific substrates associated with the PtkA-MinD complex have been demonstrated. However, their existence is probable, since MinD was shown to activate PtkA autophosphorylation (Shi et al., 2014c). The hypothesis of PtkA cycling among different activators may be an answer to the long-standing question about the rationale behind the *Firmicute*-type “split” architecture of BY-kinases. Our speculation is that *Firmicutes* employ a separate BY-catalytic domain in order for it to be able to interact with more alternative activator proteins. These interactions in turn lead to the capacity to differentially interact with, and phosphorylate, a larger number of different cellular substrates (**Figure 4**). The final consequence is an increased level of complexity in the BY-kinase-substrate network. In order to provide a definite proof for this hypothesis, our analysis will have to be extended to all known substrates of PtkA. An attempt will have to be made to quantify the *in vivo* levels of phosphorylation of all substrates in the Δ*ptkA*, Δ*salA*, and Δ*tkmA* knockouts using mass spectrometry proteomics. The hypothesis that SalA interacts with PtkA in two different modes: kinase-substrate and kinase-activator, will also have to be examined by structural analyses. While, we commit to the pursuit of these analyses outside the scope of the present report, we deem it relevant to notify the scientific community that alternative activators of BY-kinases exist, and can affect substrate specificity of these particular enzymes.

## Author Contributions

AD, AK, and LS performed the experiments. AD and IM analyzed the data. AD and IM wrote the manuscript.

## Conflict of Interest Statement

The authors declare that the research was conducted in the absence of any commercial or financial relationships that could be construed as a potential conflict of interest.
