# Supplementary material for: Substrate Specificity of the Bacillus subtilis BY-Kinase PtkA Is Controlled by Alternative Activators: TkmA and SalA
Source: Front Microbiol. 2016 Sep 26;7:1525. doi: 10.3389/fmicb.2016.01525 (PMC5035731; doi:10.3389/fmicb.2016.01525)
Supplement: Supplementary file 1 [file Data_Sheet_1.DOCX]

Substrate specificity of the *Bacillus subtilis* BY-kinase PtkA is controlled by alternative activators: TkmA and SalA

**Abderahmane Derouiche^1^, Lei Shi^1^, Aida Kalantari^1^, Ivan Mijakovic^1, 2*^**

**^1^** Systems and Synthetic Biology, Department of Biology and Biological Engineering, Chalmers University of Technology, Gothenburg 41296, Sweden.

**^2^**Novo Nordisk Foundation Center for Biosustainability, Technical University of Denmark, Hørsholm, Denmark

*To whom correspondence should be addressed. Email: Ivan.Mijakovic@chalmers.se

**Keywords: bacterial protein-tyrosine kinase, protein phosphorylation, kinase specificity, kinase activator, transcription factor**.

Running title: *B. subtilis* SalA modulates PtkA activity

**Supplementary material**

**Table S1.** List of *B. subtilis* strains used in this study.

| **Strain** | **Description** | **Origin** |
| --- | --- | --- |
| Wild type | *B. subtilis 168 trp^+^ P_r_::Neo^R^* | (1) |
| Δ*ptkA* | Δ*ptkA* | (2) |
| Δ*salA* | Δ*salA* | (3) |
| Δ*tkmA* | Δ*tkmA* | This work |
| *fatR-Step* | *fatR :: pSG1729* | This work |
| Δ*tkmA fatR-Strep* | *ΔtkmA fatR :: pSG1729* | This work |
| Δ*tkmA fatR-Strep* | *ΔtkmA fatR:: pSG1729* | This work |

1. Tanaka, K., Henry,C.S., Zinner,J.F., Jolivet,E., Cohoon,M.P., Xia,F., et al. (2013). Building the repertoire of dispensable chromosome regions in *Bacillus subtilis* entails major refinement of cognate large-scale metabolic model. *Nucleic Acids Res* 41, 687- 99.
2. Jers,C., Pedersen,M.M., Paspaliari,D.K., Schütz,W., Johnsson,C., Soufi,B., Macek,B., Jensen,P.R. and Mijakovic,I. (2010) *Bacillus subtilis* BY-kinase PtkA controls enzyme activity and localization of its protein substrates. *Mol. Microbiol* **77**, 287-299.
3. Derouiche, A., Shi, L., Bidnenko, V., Ventroux, M., Pigonneau, N., Franz-Wachtel, M., *et al*. (2015) *Bacillus subtilis* SalA is a phosphorylation-dependent transcription regulator which represses *scoC* and activates the production of the exoprotease AprE*.* *Mol Microbiol* 97, 1195-08.

**Table S2.** Primers used in this study. Restriction sites are underlined and homology with the insertion cassette is in italics.

| **Name** | **Sequence** | | **Restriction enzyme** |
| --- | --- | --- | --- |
| **Primers for Strep-tag fusion** | | | |
| *B.subtilis fatR* forward | CGGGGTACCATGATATCCGCATCCAGCAGT | | KpnI |
| *B.subtilis fatR* reverse | CCGCTCGAGTTATCATTTTTCGAACTGCGGGTGGCTCCAGGCGCCTTTTTGTGATGAATGAAGCTTAATGG | | XhoI |
| **Primers for *in vivo* mutations and fusions** | | | |
| *ΔtkmA-ext in vivo forward* | | GCTTCAATCAATGTGAACATG | NA |
| *ΔtkmA-ext in vivo reverse* | | AGGACGGGTTTTCGCGAATTT | NA |
| *ΔtkmA- in vivo* forward | | *GAGCTCGAATTCACTGGCCGT*CGATGGGAGAATCTACAAGCTTAAAAACGGGGAGTGGACATTTTGGCGCT | NA |
| *ΔtkmA- in vivo* forward | | *CGACCTGCAGGCATGCAAGCT*AGCGCCAAAATGTCCACTCCCCGTTTTTAAGCTTGTAGATTCTCCCAT | NA |
